# Everything Seems So Illogical: Constructing Missingness Between Life and Death in Israel

**DOI:** 10.1177/00302228211054317

**Published:** 2021-12-08

**Authors:** Ori Katz

**Affiliations:** 1 Department of Social & Policy Sciences, Centre for Death and Society, 1555 University of Bath, Bath, UK

**Keywords:** missing persons, ambiguous loss, death, liminality, rites of passage, Israel

## Abstract

This paper discusses the case of missing persons in Israel, to show how the category of “missingness” is constructed by the people who have been left behind, and how this may threaten the life-death dichotomy assumption. The field of missing persons in Israel is characterized not only by high uncertainty, but also by the absence of relevant cultural scripts. Based on a narrative ethnography of missingness in Israel, I claim that a new and subversive social category of “missingness” can be constructed following the absence of cultural scripts. The left-behinds fluctuate not only between different assumptions about the missing person’s fate; they also fluctuate between acceptance of the life-death dichotomy, thus yearning for a solution to a temporary in-between state, and blurring this dichotomy, and thus constructing “missingness” as a new stable and subversive ontological category. Under this category, new rites of passage are also negotiated and constructed.

## Introduction

A week after the sudden disappearance of Ehud, his ten-year-old son Michael asked his mother “So now we need to arrange a funeral?” The mother’s response was “No, what are you talking about?” But Michael insisted: “Okay, let’s wait another month and if we still can’t find him, then we will arrange a funeral.” Similarly, the mother of a missing person in Mexico asked to be given “a body, any body, so she could bury it as her son” (Ahmed, 2017). In both cases, the left-behind insisted on resolution of the mystery of missingness by using a common rite of passage, and thus to establish social death despite the absence of (indications of) biological death. While one might think that in both cases an assumption of death prevails, this study claims that rites of passage involving practices of grief and mourning are used to construct missingness as an ontological category in and of itself, consequently blurring the life–death dichotomy. In this way, those left behind fluctuate not only between different assumptions about the fate of the missing persons, that is to say, between life and death assumptions; they also fluctuate between acceptance of the life–death dichotomy (and thus yearn for resolution to a temporary in-between state) and the blurring of this dichotomy (and thus constructing missingness as a new stable category).

The subjects of this research are not the missing persons themselves, but rather those who have been left behind—the social actors who influence and are influenced by the conception of missingness. Beyond the families of the missing people, other individuals and organizations that are left behind include the police and other public officials, private investigators, social organizations and NGOs, the media, public relations firms, and the wider public who are the audience for stories concerning missing people. Through different social networks, these actors become a part of processes which give meaning to the phenomenon of missingness and develop practices for coping with the phenomenon. Missingness, thus, is negotiated and constructed through relations and interactions—making a relational component a necessary aspect of any definition of the phenomenon.

More than 4000 persons are reported as missing to the Israeli police every year. While more than 99% of them are found within a year, about 20 each year join the list of almost 600 Israelis whose whereabouts remain unknown. Whereas these missing civilians have no recognized formal or cultural status whatsoever, missing soldiers in Israel stimulate public resonance and awaken solidarity and identification (Kaplan, 2008), and the Israeli army operates a well-established and resourced unit to trace them. In contrast, a small unit in the Israeli police, with limited resources, is the only authority charged exclusively with missingness in the civilian sphere. This situation reflects the double challenge faced by the families of missing persons in Israel: coping with uncertainty, both concerning the fate of their loved ones, and concerning the behavior and actions expected of them. With this lack of appropriate cultural scripts, this paper discusses different ways of organizing the stories of missingness as narrated by the left-behind.

Following a brief overview of the literature about ambiguity at the margins of life and death and the relevant research methods, the findings will first be used to present the ontological confusion of the left-behind. The findings will then guide a discussion of how the left-behind accept the life-death distinction, using ontological assumptions (or wishes) of life and death. An examination of the construction of a new ontological category—missingness—which blurs the dichotomy and proposes a new cultural script, will follow. Finally, the findings will be directed to demonstrate how rites of passage are borrowed, reconstructed, and used in practice to enable the new status. However, this ontological category does not bring final closure for the people who have been left behind, as it is characterized by its reversibility. Hence, this process is not to be considered as linear, but rather as multidirectional and dynamic.

## Ambiguity at the Margins of Life and Death

A significant body of research produced in recent years has focused on entities that are positioned at one of the two edges of life. The viability of these entities is constantly negotiated by different actors; often in light of new medical technologies, life-extending technologies on one side and reproductive technologies on the other. Thus, new social and cultural contexts can create new kinds of “persons,” and new categories of “life” (Kaufman, 2000). The ontological status of these entities is determined by the viability attributed to them in different relationships and settings (Bassett, 2015; Behuniak, 2011; Howarth, 2000; Rimon-Zarfaty et al., 2011; Svendsen, 2011).

Kaufman and Morgan (2005) describe some of these situations as liminal. Sometimes, this liminality is chronic, such as in the case of permanently comatose individuals, who can only leave this state through death (Kaufman, 2000). However, Bird-David and Israeli (2010) claim that the vegetative state disrupts the common categories of life and death, such that it can no longer be described as a liminal state falling “betwixt and between.” Rather, it is a relational state, described ad hoc according to relations, and lacks a single status. According to Bird-David and Israeli, caregivers move between the two processes, of “emptying” their patients’ personhood and “repersonifying” them.

Many of these in-between states are temporary, despite the fact that they challenge the binary distinction between life and death; this liminality only postpones the return to the familiar and clear distinction between these two basic human states. And yet, many scholars have recently discussed entities whose state is not definable as in-between but rather destabilizes the hitherto settled notion of personhood. This literature, sometimes defined as “posthumanism” (Sharon, 2014), shows how new technological developments, such as eugenics and cybernetics, can construct new kinds of “humans.” Thus, death might no longer be perceived as “natural.”

But, while advanced technologies keep challenging notions of personhood, humanness, life, and death, missingness shows how an ancient category, which is not based on technology (on the contrary, technology aims to diminish this phenomenon by tracing missing persons easily), similarly confronts these notions. Several terms have been employed to try and conceptualize this phenomenon, including “suspended death” (Kaplan, 2008), “living in limbo” (Holmes, 2008), and—probably the most influential—“ambiguous loss” (Boss & Yeats, 2014), referring to a loss that remains unclear and without resolution. The concept refers to two kinds of loss: the loss of a loved one who is physically absent but psychologically present, as is the case with missing persons; and the loss of a loved one who is physically present but psychologically absent, as with people suffering from dementia (Boss, 1999, 2002, 2007, 2016). In this sense, ambiguous loss is a unique loss, due to the intersection between grief and hope (Boss, 1999, 2016; Heeke et al., 2015; Hollander, 2016; Holmes, 2008; Lenferink et al., 2018). Wayland et al. (2016) expanded this theoretical discussion, describing how the relatives of missing persons fluctuate between hope and helplessness even as they grieve the physical loss of their loved ones.

With ambiguous loss, accurate information is usually unobtainable. The relativity of truth that accompanies this lack of clarity leads people who are experiencing ambiguous loss to search out meaning for the situation. Thus, Boss (2007, p. 106) suggests, “instead of the usual epistemological question about truth,” scholars should remember the dynamic relational character of ambiguous loss, and the paradoxical possibilities of change inherent to states of ambiguity. By examining the dynamic role of life and death perceptions among those left behind, this study accepts Boss’ call.

However, while this theory is very influential—pointing out, for example, the type of loss, and not just the type of grief (Boss & Yeats, 2014)—aspects of the social categories that may be constructed following ambiguous loss remained under-researched. Thus, the aim of this paper is to add another facet to this discussion by examining the construction process of “missingness” and its subversive potential.

This study focuses on the Israeli context, which illustrates how the state uses the ambiguous zone between life and death to its own benefit, by using the missing soldier figure. Missing soldiers serve as a bridge between the collective life and the collective loss, operating “as a literary solution to the acute paradox experienced by a society that sacrifices the lives of its sons in the name of collective ideals and at the same time assigns a central value to the sanctity of life” (Kaplan, 2018, p. 194). The missing soldier becomes a totem for the national community, and is thus constructed as a collective dead-alive figure. In this sense, missing soldiers do not threaten the life-death distinction; on the contrary, they strengthen it, by creating a mythical bridge personifying collective solidarity (Kaplan, 2018).

But contrary to the collective perception of living-dead missing soldiers, missing civilians are perceived as individuals who are neither alive nor dead. However, this unrecognized category has the potential to become a subversive one. The Dirty War of the 1970s and early 1980s in Argentina, where over 30,000 people were “disappeared” by the military regime, is a good example. The recurring protests of the families of the disappeared, mainly the “Madres of the Plaza de Mayo,” rejected all common scripts and refused to reconstitute the disappeared as dead (Edkins, 2011). By doing so, the people left behind refused to “heal” their trauma, instead insisting on preserving the traumatic impact in order to challenge the political order: “Let there be no healing of wounds. Let them remain open. Because if the wounds still bleed, there will be no forgetting and our strength will continue to grow” (Holst-Warhaft, 2000, p. 117). The disappeared kept their agency through those left behind and continued to shape politics and debates about grief and about the relationship between the state and its citizens. The trauma was intentionally placed in the public sphere, to fight “order that relies on tidy categories, stories of continuity, and the exclusion of anything or anyone that does not fit” (Edkins, 2011, p. 156).

Continuing this line of thought, missing civilians in Israel “do not fit.” They may go unrecognized, without any cultural script of missingness. But otherwise, as the Argentinian case illustrates, they may become the subjects of a new and subversive social category. This paper seeks to examine the constant movement of missingness between a temporary and perpetual category; between accepting the life-death binary distinction and challenging it. Mostly, the perception of missingness begins with temporariness and the aspiration for an ontological solution; continues with the breakdown of this expectation (entailing the breakdown of the life-death distinction); and ends with constructing missingness as a category in its own. Nonetheless, this movement is not necessarily straight, deterministic, or linear.

## Methods

This paper is based on a research study conducted between 2015 and 2019, which explored the processes of constructing narratives relating to civilian missingness in Israel. The paper’s premise is that the ambiguity that attaches to the phenomenon of missing persons pushes the people who have been left behind into deconstructing and reconstructing their personal, familial, and social narratives. This entanglement is complicated by the lack of a clear ending to the story and the lack of cultural scripts of civilian missingness. Thus, the process of constructing narratives is placed at the center of the analysis. The object of the analysis is, therefore, the construction of the event, and not only its narrated outcome.

To examine these narrative construction processes empirically, I conducted a narrative ethnography: an approach in which the researcher seeks to be present at different stages of the narrative construction process, with an acute awareness of the myriad layers of social contexts (Gubrium & Holstein, 2008). This methodology does not aim to trace one’s story; rather, the objective is to follow the stories that others tell about the event in question, about themselves, and about their common social worlds. In this way, one can identify different possibilities for the process of constructing a narrative—but without the pretension of mapping a particular story and its evolution over time.

A significant part of the narrative ethnography conducted in the course of this research is concerned with *Bil’adeihem* (“without them”)*,* an association of families of the missing, established a few months after Daniel Minivitzky’s disappearance in October 2014. It was the first time that the families of missing persons in Israel had gathered in order to raise public awareness about the phenomenon of missing persons, and to lobby the state to improve its protocols for searching for missing persons and supporting their families. I participated in activities of the association from its establishment onwards: meetings, public events, and lobbying activities supporting the regularization of the status of missing persons in Israeli law. I also took part in other events, such as “searches”—a term describing different kinds of practices implemented by those left behind to find answers to different questions about missingness (Katz, 2020). I also carefully followed both mainstream media and social media coverage of occurrences of missingness, and analyzed relevant texts and documents.

In addition to this, I also conducted 33 in-depth interviews. 22 of these were in-depth interviews with family members of long-term (between one and more than 40 years) missing persons. Sixteen of the interviewees are women, and 6 are men (of their missing relatives, the distribution was the exact opposite: 16 men and 6 women). A further 11 interviews were conducted with other actors, to conceptualize different perspectives about missingness. Three of these interviewees were police officers; three ran voluntary associations; the others included a private investigator, an employee of the national forensic institute, a journalist, an activist with *Bil’adeihem*, and a public relations professional advising the association. Informed consent was obtained from all the participants, and pseudonyms have been used throughout the text. The research was given ethical approval by the ethics committee of the Ben-Gurion University of the Negev.

Typologies of circumstances of missingness are intentionally avoided in this paper. Missing persons’ researchers (e.g. Biehal et al., 2003) often seek to create such typologies that categorize missingness, reduce its ambiguity, and frame it within certain assumptions. Contrary to this, this study joins Edkins’ (2011) claim that the only thing that missing persons have in common is that someone is looking for them. Thus, this research devotes itself to the ambiguity and uncertainty that construct and operate missingness. Moreover, while much scholarly attention is given to missing persons in the contexts of military (“missing in action”), natural disasters, and forced disappearances, the participants of this study do not suggest that their loved ones have gone missing following any of these circumstances. The category of civilian “missingness,” as discussed in this paper, is therefore often perceived as a problem of individuals, rather than a collective problem (Katz, 2020).

^Findings^

### The Ontological Confusion of the Left-Behind

Missingness is characterized by ambiguity and uncertainty. To be precise, it is almost impossible to imagine a harder and stranger mystery than one that shrouds the most basic states of existence—life and death. Shuki Minivitzki, whose son Daniel has been missing since 2014, asked during an interview with a journalist: “How is it possible to live under this uncertainty? You either have a child—or a corpse” (Shir, 2016). Clearly and concisely, Minivitzki expresses the binary essence of human existence, which allows for only two possibilities. But ever since his son’s disappearance, this essence has crumbled; today, there is no child and there is no corpse.

In this way, the ones who have been left behind find themselves confused, embarrassed in the face of this unsolved mystery. Minivitzki names the two scripts that one may think of, while a third script is actualizing before him. This is how the binary distinction between life and death collapses, according to Orit, speaking a few months after her brother’s disappearance:

*We remain optimistic. Otherwise, I think we wouldn’t keep searching* […] *But we are also realistic, because I really don’t know what happened to him. At the beginning, we were saying ‘What do you think? Where is he?’ Now I really, personally, don’t know what to think. Every possibility doesn’t make any sense to me. Even if something happened to him, God forbid, we’ve been told that they would find, like, a corpse or a piece of cloth, or a bag. But they haven’t found any of these. So everything seems so illogical.*

Orit describes how the assumption of life falls apart (“we’re realistic”), but the assumption of death is yet to be established (since no corpse, no piece of cloth, no bag have been found). In this manner, the known rules of this world cannot govern missing persons, and those left behind are not able to think about them using questions such as “Where are they?” Hence, each and every script “seems so illogical,” and the binary distinction between life and death is challenged.

This third script is characterized mostly by what it does not have: the lack of knowledge, of facts, of witnesses. While life has a very clear definition, and declarations of death follow detailed protocols (even though this may be challenged in certain situations), missingness provides a space defined by absence. Despite its absent character, it problematizes the life-death binary dichotomy, making this basic distinction much more difficult. As Amir, whose mother, Yaffa, has been missing for 20 years, recounts: “Each claim you could make about her being alive, and each claim you can make about her being dead, I can contradict and confirm.” Amir’s words reveal how the missingness space has a central feature despite its uncertainty and ambiguity: it is reversible. Moreover, the people who have been left behind have an active role in the construction of missingness and its movement. In the ontological fluctuation that they experience, they might construct missingness as a temporary category and aspire for an ontological solution, or construct it as a perpetual category that is neither life nor death.

### Accepting the Dichotomy: Holding on to the Hope for Life

The missingness space provides some liberty for the people who have been left behind to construct their ontological assumptions about their loved ones. If reality is so strange (“everything seems so illogical”), it is no longer reasonable to calculate probabilities regarding this reality. For many, if it is illogical to assume that the missing person is dead (as it is also illogical to assume that he or she is alive), it is better to hold on to the life assumption. “I am willing to rely on anything that would indicate that Modi is alive,” said Miri, whose brother has been missing since 2010, in an interview with a journalist (Ariel Amir, 2016). Miri commits her thoughts to the life assumption—assisted by any indication, real or imagined, to construct her hope. Yossi Ya’acobi, whose daughter Adi has been missing since 1996, described his construction process in another interview with a journalist (Kotas-Bar, 2013):

*It is as if you have a sick child* […] *and everyone says, ‘That’s it. There is nothing else to do,’ and only one doctor says ‘Come, I will save your child.’ Clearly, you will go to this doctor and not to those who say that there is no chance. I am her father, and I wait for her.*

The decisive issue of the ontological question for Ya’acobi is not the reality, nor any kind of probabilities calculation, but rather his relationship with the missing person. His explanation for embracing the life assumption is simple and honest: “I am her father."

Different agents help in the construct of the assumption of life. If the life–death dichotomy dominates, and as long as death is still perceived as a bad scenario, the families of the missing keep hearing others’ wishing for them to receive “good news.” In the absence of a clear social category of missingness, they frequently feel obligated to keep on searching for their loved ones as though they are alive. This approach is also prominent in *Bil’adeihem* activities. One of the association’s strategic goals is to highlight success stories in locating missing persons. For example, in one of its meetings, one of the activists said on the stage: “I can offer a scoop for you: this association, since it became active, has managed to trace two missing persons.” Another activist gave more details: “We found a missing person through publications and advertisements, and it is important to make it public. He had lived for more than 4 years under the radar and he was found alive, homeless.” These stories frame missingness as a temporary category, and the association as one which has the ability to bring about the hoped-for resolution. They highlight the importance of making success stories public in order to show that missingness stories do not remain without closure forever. The association’s Facebook page also focuses on the need for closure, presenting potential solutions as something that “must happen” and that “will come for sure”; stating that “it is impossible to stop believing.” Another success story’s post ended with these words: “Share this post. There is hope!” These messages of hope aim to strengthen not only the assumption of life but also acceptance of the binary distinction between life and death.

### Accepting the Dichotomy: The Death Wishes Among the Left-Behind

Sometimes, the meaning of the wish for “good news” is changed. The absence is so unbearable, that the yearning for something material brings many to compromise: if the missing person is not going to be found alive, let her be found dead, and let her have a grave. Lowering his voice, as though he was saying something forbidden, Amir told me: “Everybody wishes to trace their loved one alive and to go through this path of agony. But […] deep inside my heart, I wish to finish this story, I want this stone to fall off my heart.” Amir presents his death wish (for his mother) as situated alongside the hope for life. He truly wishes to trace his mother alive, and will even go through a “path of agony” to this end. But simultaneously, a death announcement wish is developed quietly. News about his mother’s death would now bring relief to him.

This death wish is expressed by many family members of missing persons, almost always quietly and with uneasiness. This was, for example, expressed by Naomi, whose grandfather has been missing for five years:

*In January, my friend’s father passed away* […] *I was jealous.* […] *They had time to separate, they had time in hospital, it’s okay, everything is okay, he was buried, you know where his grave is, you know what happened to him, you have separated.* […] *This is the natural thing. It’s all for the best.*

Life alongside missingness is so difficult that death becomes something positive, “for the best.” Death is framed by Naomi as “the natural thing” as opposed to the unnatural missingness, which breaks the longed-for binary between life and death.

Vered, a teacher, whose father has been missing for four years, takes this perception of death one step further, framing it as “fun”:

*I lost my student, who passed away.* […] *So I said ‘She was suffering, and she is no longer suffering. Look how fun it is. She is no longer suffering!’ My attitude to grief and to death has changed. It has an ending!*

The unexpected formation of missingness has changed Vered’s perceptions of grief, life, and death. Once another category, missingness, manifested, death was no longer so threatening.

Hanna, whose ex-husband, Yuval, has been missing for 20 years, describes their daughter’s perception of death following the passing of her friend’s mother: “The first thing [my daughter] said was ‘I am so jealous. […] it’s so fun to know where your mother is and to have a grave.’ Do you get how psycho it is?” In the “natural” world, as Naomi describes, that has a clear distinction between life and death, the words of Hanna’s daughter are “psycho.” But in the world of the ones who have been left behind, death is perceived as good news due to its implications: a closed story and a grave.

But the statements of envy are not always declared quietly. In a *Bil’adeihem* meeting, Shimon, whose father has been missing for 20 years, cried and shouted at an Israeli Police representative in attendance:

*I’m getting old. I don’t know what will happen tomorrow. I must bring a closure to dad’s story. I put my head on the pillow at night and don’t know my father’s whereabouts! I am jealous of children who bury their father. Look how low I got, Good Lord!*

Even if Shimon was feeling alone with his envy, as many of my interviewees felt, I am sure that many people in that room nodded when hearing his outburst. Thus, *Bil’adeihem *meetings became an arena for dimming the loneliness of the left-behind; in its place, the missingness category became clearer, finding its location between life and death and constructed as a stable category of its own.

## Blurring the Dichotomy: Missingness as a Category of its Own

Aharon, whose father has been missing for six years, describes the construction process of missingness as a category on its own:

*When someone dies, it hurts. You receive the punch, assimilate it for a while, and then go on with your life. Here, you cannot do it. For a long period, you assimilate one more percent, and one more and one more, and it becomes longer, harder, and of course uncertain. I cannot explain it, but it is problematic.*

Like the loading process for software or an internet page, Aharon describes the progress as a percentage, signifying the assimilation of moving from one status to another. Even though in the case of death it takes “a while” for the left-behind to assimilate the new status of their loved one, missingness forces a much longer period of assimilation. And yet this process happens; it does not stop, but rather progresses “one more percent, and one more and one more.” But for as long as the missingness persists, the percentage will never reach 100%. The people who have been left behind could be almost certain that their loved ones are dead, but the one percent that makes this process complete is still missing. This is the uncertainty that makes missingness reversible. Aharon, who stated throughout the interview that he had moved on with his life, still faces this unlikely reality, which he “cannot explain.”

Often, the construction of missingness as a category also indicates the assumption that the missing person will never be found. Thus, the missingness category fulfills, at least in part, the age-old longing to fight death, deny death, and attempt to postpone death for as long as possible. Yet, it does not provide eternal life, not as people have imagined immortality throughout history. Indeed, it might only help to construct a space for ontological imagination. This is, for example, what Dennis said five years after his 76-year-old grandfather disappeared (Vardi, 2017):

*We got used to this situation. And you know what? It might be good. Because in my memory, I remember him alive and smiling with the family. And I may think he is still alive somewhere not very far away, and maybe he even feels good. After all, we have not seen him dead and we were not notified that he is dead. And maybe this even holds grandma alive.*

Dennis and his family “got used” to life alongside missingness, and they have even found its advantages: it preserves the memories of living by not providing any single memory of death, and thus provides flexibility when imagining the missing person’s fate. It is possible to imagine him alive and even to think he “feels good”; this imaginary scenario might help some of the left-behind, like Dennis' grandmother, to stay alive. As long as there is no clear notification of death (“We have not seen”; “We were not notified”), his grandfather remains located in this unique space between life and death.

The understanding that the missing person might never be found is interlinked to the families' wish to “keep living.” Once families of the missing acknowledge that it is not necessarily a temporary state, they are advised to “return to their lives,” both by scholars (such as Boss & Yeats, 2014; Lenferink et al., 2018) and by professionals, such as Avi Zelba, a criminologist invited to speak at the formal launching event of *Bil’adeihem*: “The secret, and forgive me for being so harsh, is that you may never know what happened. […] So I can only say what I think, keep on with your lives.”

These academic and professional experts of missingness tie up the construction of missingness with the personal and familial decision to keep living. This study claims that this process is also tied up with the blurring of the distinction between life and death. The ontological assumption about the missing person’s fate is interrelated to the coping mechanism of the people who have been left behind. As long as missingness is perceived as a temporary state, there is a yearning for an ontological solution, and the dichotomy between life and death is accepted. But once missingness is constructed as an ontological state of its own, such that this dichotomy is challenged, the left-behind may reconstruct their lives and their relationships with their loved ones. This is, for example, what Orit, who became engaged a short time before her brother’s disappearance, said:

*At the beginning it was very hard and I said I don’t want to get married. […] Until we finally realized that it is enough, we want to get married, we want to keep our lives. […] We are realistic, we really can’t say for how long it is going to last. And what if it lasts long period? So, we won’t get married?*

The dilemma about Orit’s wedding became a negotiation about her brother’s ontological state. Due to the lack of cultural scripts relating to missingness, this negotiation was needed to make a decision about the wedding. And once the family “finally realized” their situation, once they became “realistic,” they could decide to get married without further waiting. Yet, Orit expresses her “realism” by saying that this situation might last for “long period” of time. She does not say out loudly that her brother might never be found. However, “long period” is enough for challenging the temporary assumption, and for blurring the life–death dichotomy. Missingness is always reversible, and it does not have the ultimate-ending quality that death has (Katz & Shalev Greene, 2021). But, it still might be constructed as a category of its own.

Seemingly to the contrary, families of the missing who “return to life” may also be tied up with accepting the life–death distinction and not blurring it, through request for a formal declaration of death. Israel’s Death Declaration bill permits courts to formally determine a missing person’s death seven years after disappearance. Yet, the law indicates how different this status is from other deaths, by stating that the petitioner must publicly post a message “that provides, to the court’s opinion, sufficient chance for the presumed-dead person to notify [the court] that he or she is alive.” The bureaucratic way out of missingness is thus possible, but throughout the years, only a few families of the missing have chosen it. This possibility, the law says, is reversible, and hence it is not a biological death but a bureaucratic one. It would thus not provide the necessary completion of Aharon’s percentage, nor achieve the final ontological status of death. *Bil’adeihem* has sought to establish another bureaucratic solution, through proposing a law to regulate the status of missing persons in Israel. In these meetings they raised several juridical possibilities, such as “a missing certification” and “legal person’s cancellation.” Thus, it seeks to establish a third formal ontological state, other than “alive” or “dead,” recognized by the state.

## Blurring the Dichotomy: Constructing Rites of Passage and Rituals

In order to establish missingness as a new social category, rites of passage are needed; symbolic practices that would end the liminality and the perception of temporariness that characterize the ongoing waiting for a solution. As long as there are no rites of passage for the people who have been left behind, missingness cannot be constructed as a stable category and the life-death dichotomy is still acknowledged. Therefore, an important aspect of the missingness construction process is the construction of new rites of passage. This kind of ceremony symbolizes a closure, an ending to the liminal state, enables rituals for practicing the new status of the left-behind.

Sitting Shiva, the Jewish period of mourning, is a ritual widely borrowed by the people who have been left behind. Varda Minivitzky, whose son Daniel has been missing since 2014, described in an interview the first week of searches: “It was like Shiva, people were coming and sitting with us. […] It was good for me because I was able to tell stories about Daniel over and over again” (Shir, 2016). Even though sitting Shiva for a deceased son is one of the hardest rites of passage that exists, it provides options to meaning making and to storytelling. The ritual confers so many advantages that it gave Varda a “good” feeling.

Naomi, whose grandfather has been missing for five years, describes the practical implications of borrowing this ritual:

*We decided to try and go back to our daily life. Like, this week of searching was kind of Shiva, and now we can go back to our daily life. Maybe to keep searching on Fridays and Saturdays a bit, maybe trying from some other directions. We didn’t say we have stopped searching, but it gave us this legitimacy to go back to our daily life. After two or three months, we just stopped [searching].*

Thus, the sense of sitting Shiva, sometimes constructed retrospectively, did not bring Naomi’s family to an assumption of death; but it provided a rite of passage, which led them to their new status. Eventually, it provided legitimacy they needed to stop searching and to go back to their daily lives, which from now on is lived alongside missingness.

Other than Shiva, cemeteries are a site of symbolic practices used by the left-behind. Since Michal’s father’s disappearance, she visits her grandparents' graves twice a year: on her father’s birthday and on the day of his disappearance. “There is a small space in between the graves,” she says, “so I’m saying it is dad’s part.” Inspired by common ceremonies of memorialization, Michal constructs continuing bonds with her missing father. Doing it at the graves of her father’s parents, she creates an intergenerational encounter between three ontological states: the living, the missing and the dead. The relationship between the living and the missing is well exemplified also in the story of 6 years old niece of Vered, whose father has been missing for 4 years:

*On my father’s first birthday after he went missing, [my niece] took a flower, dug a hole in the ground, and put it there. […] My brother asked her why she is doing it, and she said ‘because I have no other place to wish grandpa a happy birthday.'*

Both Michal and Vered’s niece, as well as the 10-year-old Michael quoted at the beginning of this paper, illustrate the flexibility of children with constructing and reconstructing new rites of passage, and cultural scripts, followed by establishing new social category.

Memorial days suggest more practices to do that. As Amir recounts: “Once a year, on the day of my mother’s disappearance, my wife and I go to a café, eating, drinking and talking. It’s important to her since she doesn't know [my mother].” This annual conversation is used to introduce Amir’s mother to his wife. Doing it on the day of her disappearance adds a ritualistic aspect to this event, borrowed from memorial days, constructing a new ceremony to cope with missingness.

The public relations firm that works with *Bil’adeihem* to raise public awareness of missingness aimed to declare a public day for civilian missing persons. This step is not only designed to help the families of the missing cope with missingness, but to construct missingness as a collective recognized category. By borrowing symbolic ritual such as national memorial days, they do not only deal with private or familial grief, but with national grief too; they seek to reproduce not only a well-known category, but one with high cultural prestige.

However, these new rituals also have a problem. While death fills its ceremonies and rituals with ultimate certainty and non-negotiable terms, missingness is characterized by uncertainty and ambiguity. Thus, it is much more difficult to translate this state into symbolic and ritualistic practices. The missingness category places a challenge to the life-death dichotomy, but the third ontological state it offers is much more ambiguous and reversible. Therefore, it is sometimes deficient as a rite of passage, and sometimes feels like a constant and endless rite of passage. This is best illustrated by Orit, whose brother has been missing for almost three years: “You cannot even sit Shiva, it’s like an endless Shiva, kind of.” All at once, missingness is impossible (“you cannot”), inevitable (“endless”), and undefinable (“kind of”). It is constructed as a temporary category, a perpetual one, and everything in between.

## Discussion

The empirical analysis of this paper begins by depicting the ontological confusion experienced by those left behind, followed by their yearning for a solution, holding on to the hope of life on one side and a death wish on the other. While this yearning reflects acceptance of the life/death distinction, the construction of missingness as a social category challenges this dichotomy. Thus, alternative rites of passage must be constructed in order to accommodate this disjuncture. However, this seemingly linear development is actually a multidirectional one. Life in the shadow of missingness creates a relational and dynamic movement, one that convulses the life-death dichotomy. This movement is based on the ambiguity and uncertainty inherent to missingness, along with the paradoxical potential for change that it creates (Boss, 2007); it reflects prevalent assumptions and cultural scripts, and simultaneously constructs new ones.

The life–death dichotomy remains solid long after other categories have become more liquid than ever. Even the rites of passage used by the people who have been left behind are taken from practices of mourning and grief, making this coping mechanism seems like the result of a strong assumption of death. However, missingness is reversible by definition, and it does not matter how little the percentage left to complete Aharon’s imaginary percentage counter. In this sense, the rites of passage would never make missingness as ultimate as death, or as certain as life. Using rites of passage and other practices and narratives, missingness is constructed as an ontological category of its own. It does not suggest a new category of “life” (Kaufman, 2000), but a new category of human state, positioned between life and death, characterized by its reversibility. It is therefore not a temporary category, yet it is also not a permanent one.

In this fashion, and even though rites of passage are used, missingness is not a liminal state, nor an end to a liminal state. Rather, it is a relational state, determined by interactions and negotiations. However, these negotiations take place quietly, only among the few actors who have been left behind. When someone goes missing, the families need not only to trace their loved ones, but also to look for cultural scripts, guiding them in their efforts to search, to cope, and to construct a narrative under circumstances of uncertainty and ambiguity. But as they find out quickly, they face a lack of recognition regarding civilian (as opposed to military-related) missingness.

This lack is not necessarily coincidental, but rather characterizes ambiguous loss (Boss, 1999, 2002). The dead–alive figure of the missing soldier in Israel reveals how the state strives to control the boundary zone between life and death, and even to use it to its own purpose (Kaplan, 2008, 2018). The unrecognized phenomenon of missing civilians can be seen as another example of the modern effort to reduce the subversive potential of this boundary zone. However, this study claims that this potential manifests precisely as a result of ambiguity and the attendant absence of cultural scripts. Without such cultural scripts, those left behind can construct their own narrative, followed by a construction of the new and subversive social category of civilian missingness. Under the missingness category, the missing person becomes a “zombie,” a rebellious figure threatening the perception of life and death as dichotomous categories.

*Bil’adeihem* has had a huge impact on the people who have been left behind, and on the occurrences in the boundary zone between life and death. While the state offers the possibility of seeking a declaration of death from the authorities, *Bil’adeihem* tries to establish a recognized status of missing persons through the law. In a way, and despite the differences, these efforts are equivalent to the “Madres of the Plaza de Mayo” and their refusal to reconstitute the disappeared as dead (Edkins, 2011). Whether the families of the missing in Israel do this intentionally or not, their process of constructing missingness, as in the Argentinian case, might create a movement between the so-called solid categories of life and death, and a third ontological state with a subversive potential.

The construction of missingness as a social category may also have an impact on those left behind coping possibilities and strategies. As civilian missingness is increasingly recognized, by both professionals working with the families and other actors, the families of the missing are able to construct their own narratives, whether they wish to hold on to the life assumption, to presume their loved one is dead, or to construct missingness as a status that is here to stay. As this is a preliminary research study focusing on the Israeli case, future research is needed to consider the relationship between social recognition (and perhaps formal regulation) and the coping practices of families of the missing. A comparative research perspective would be useful in this respect.
